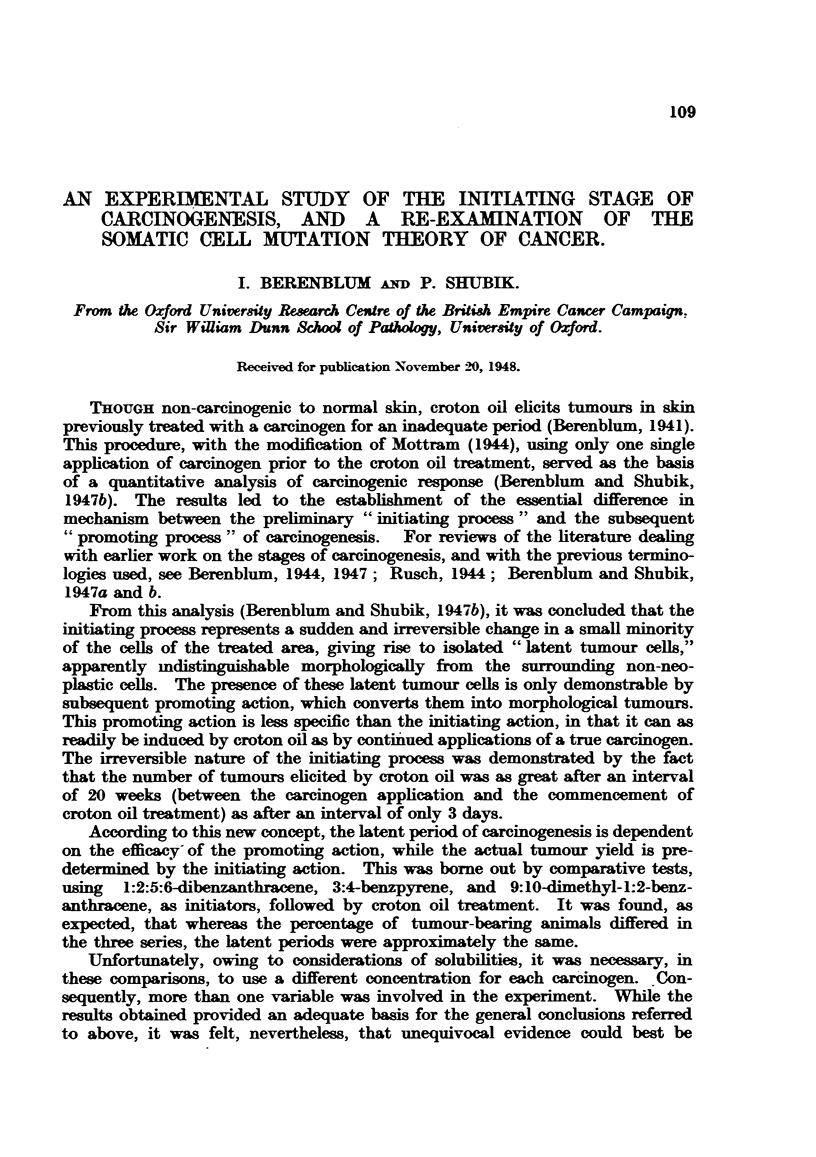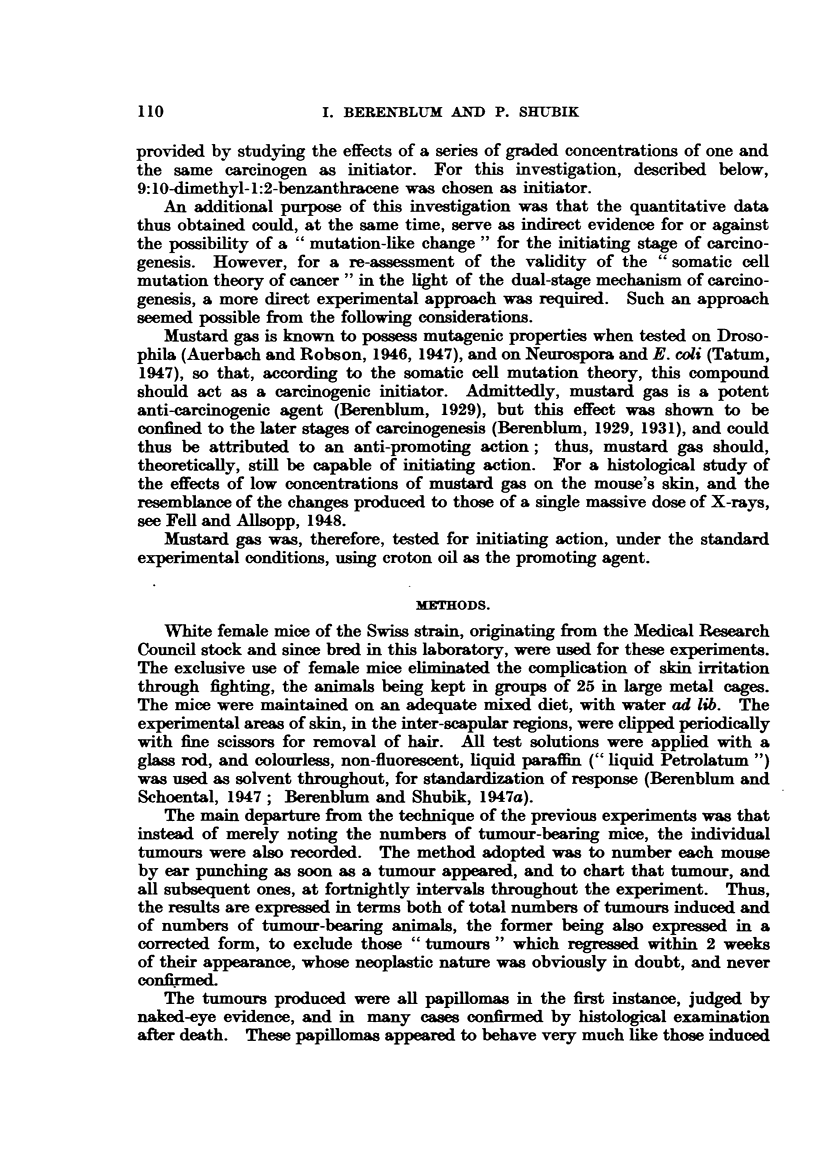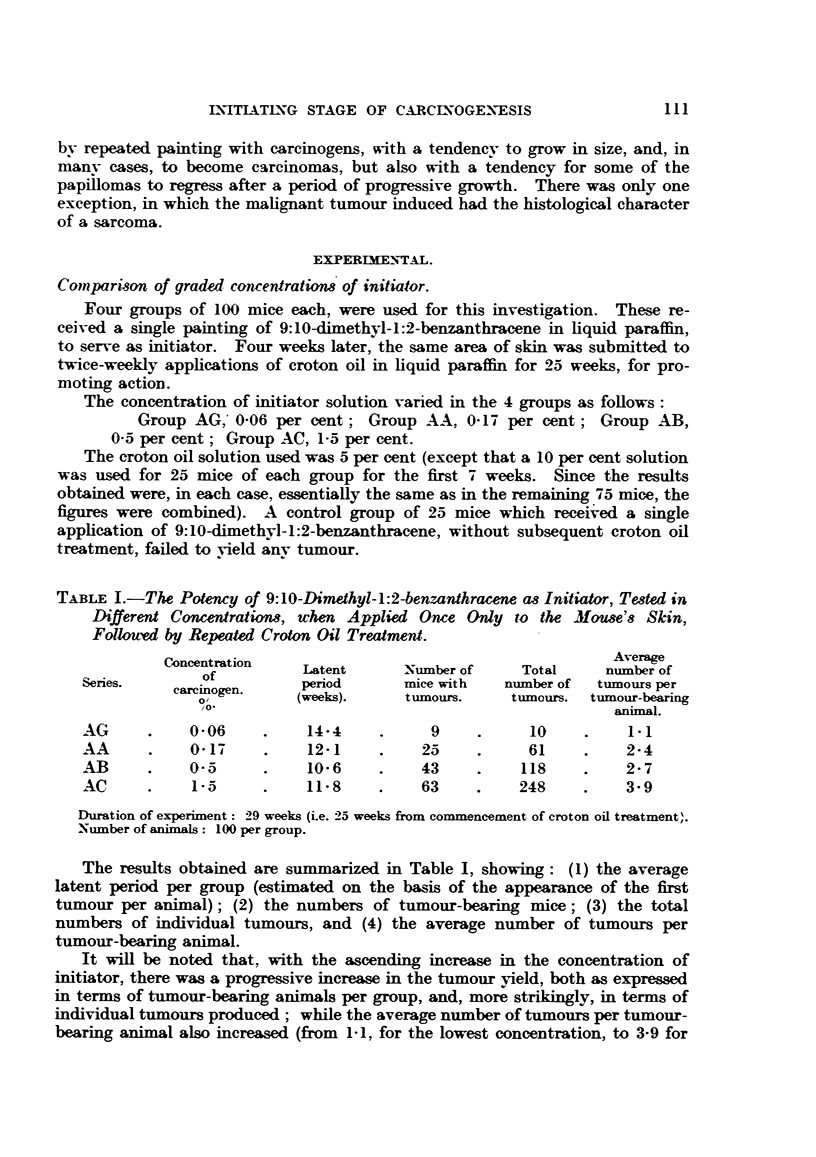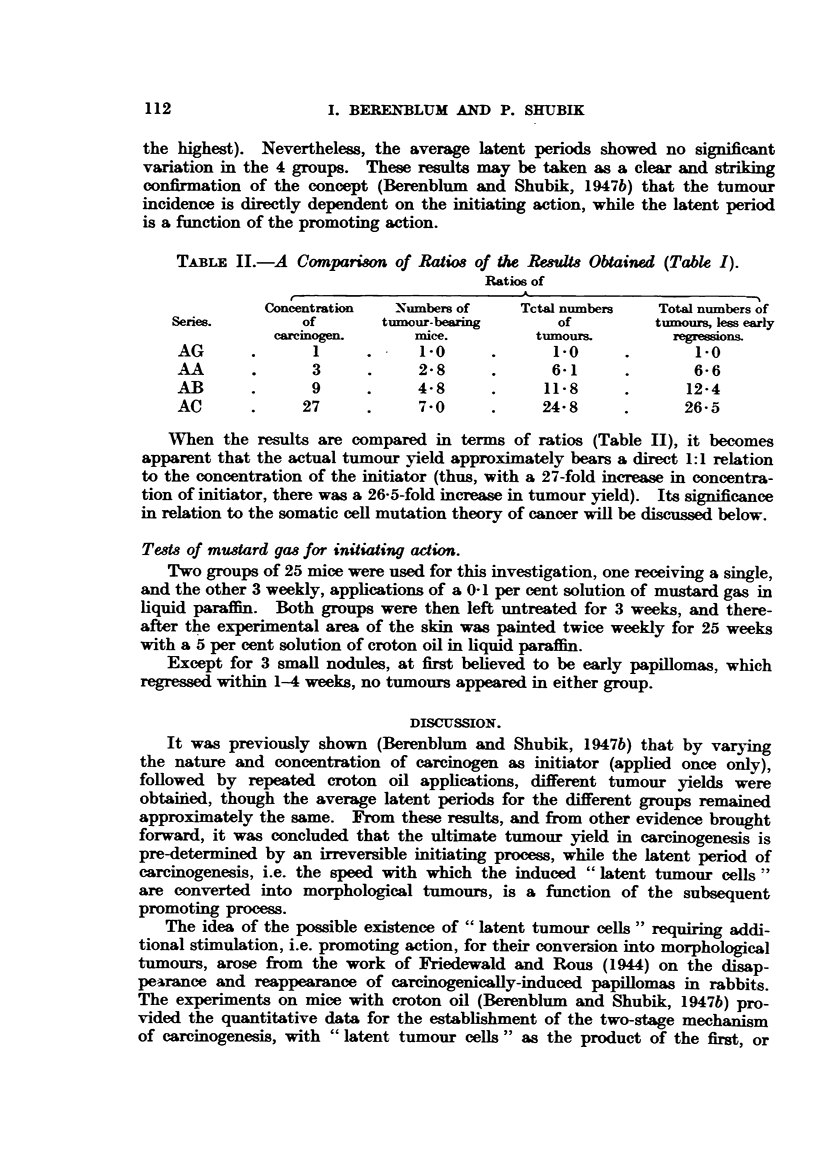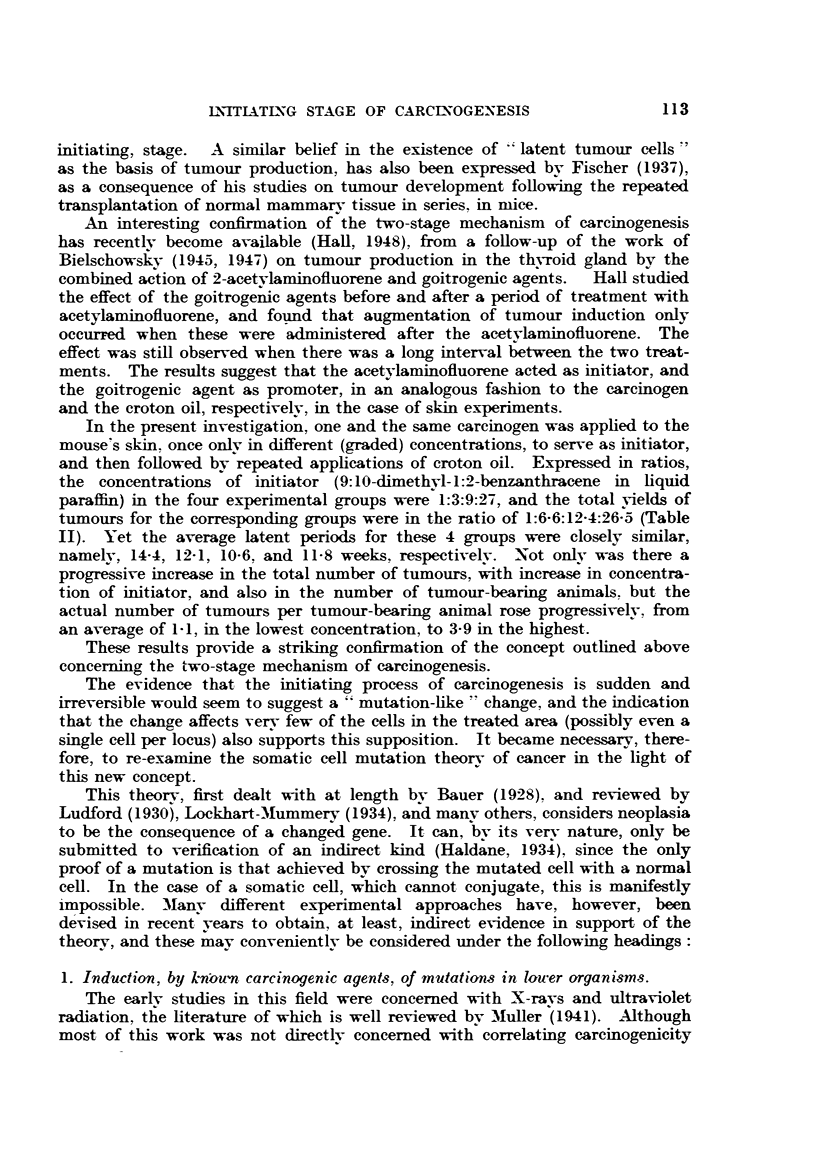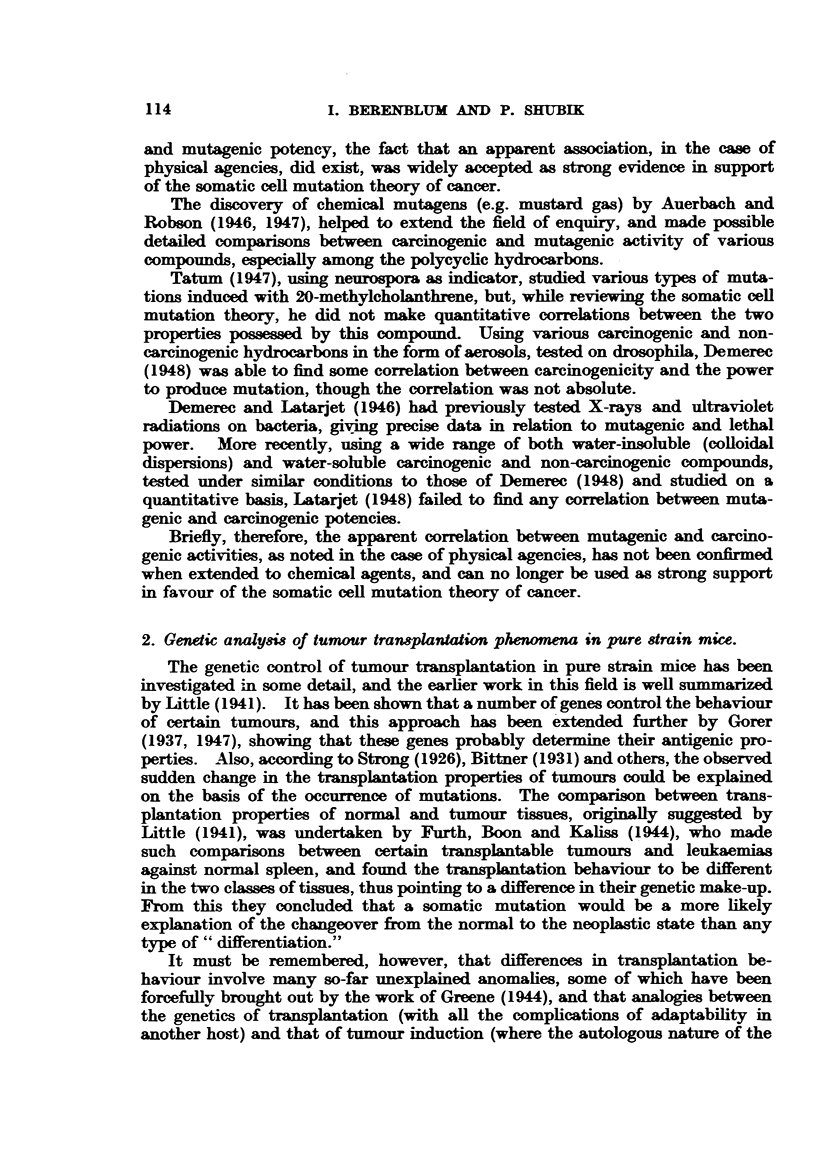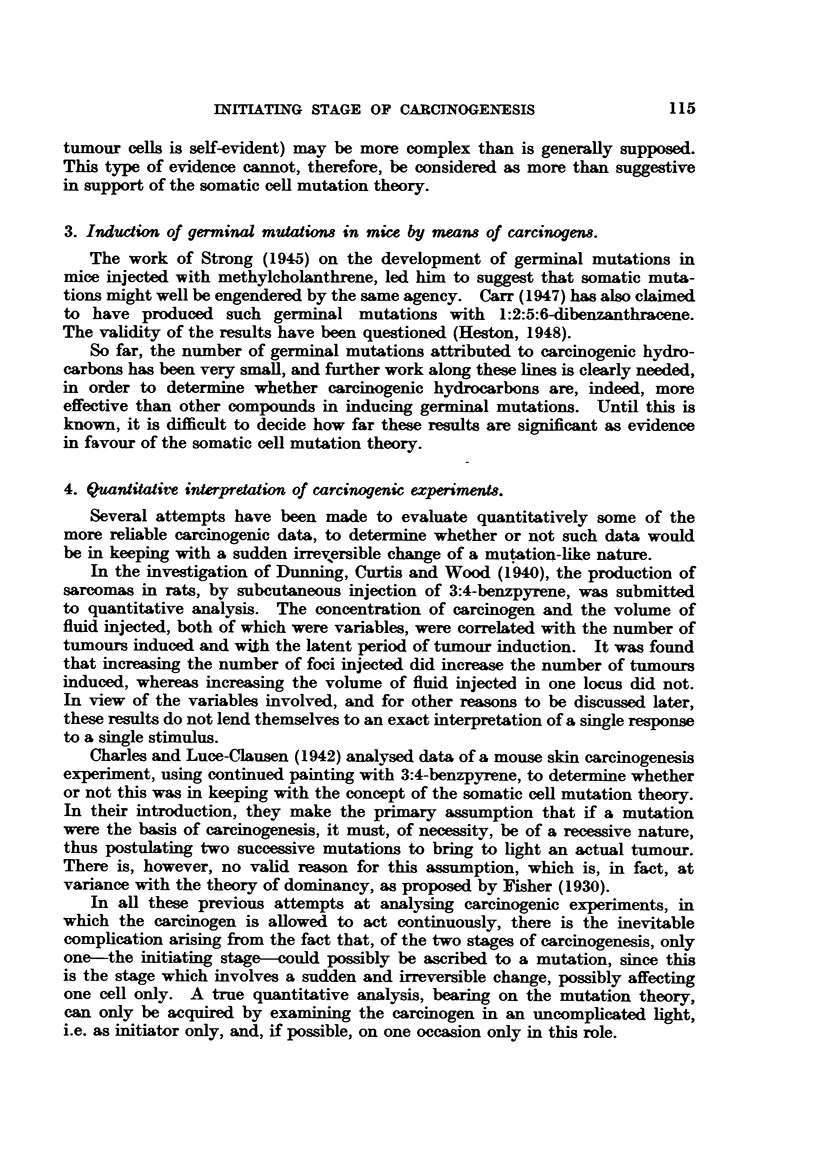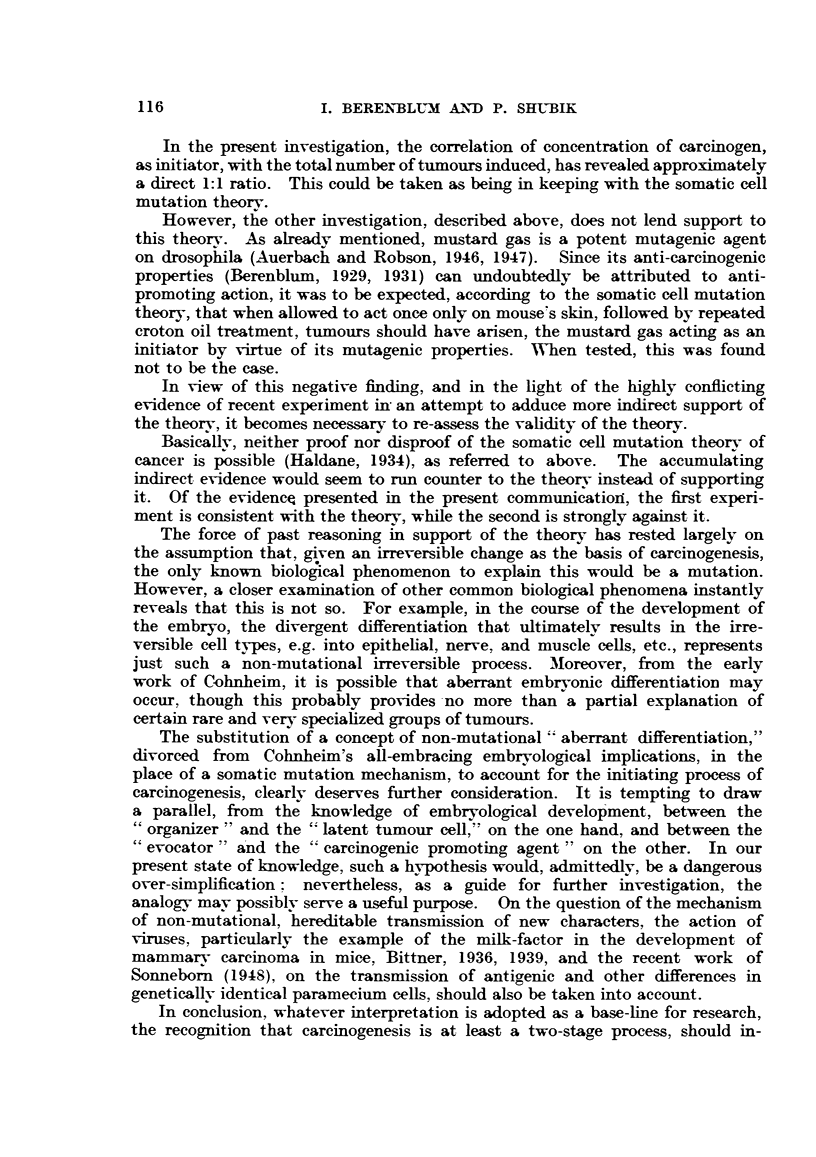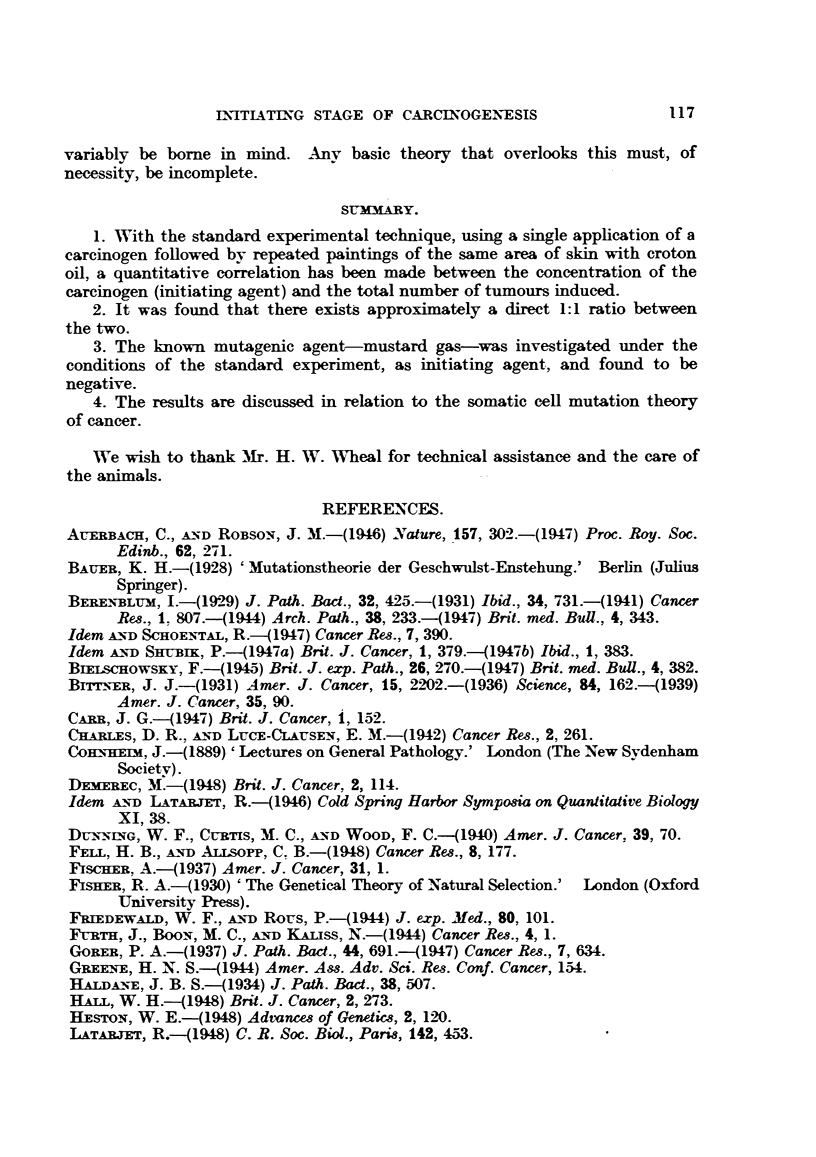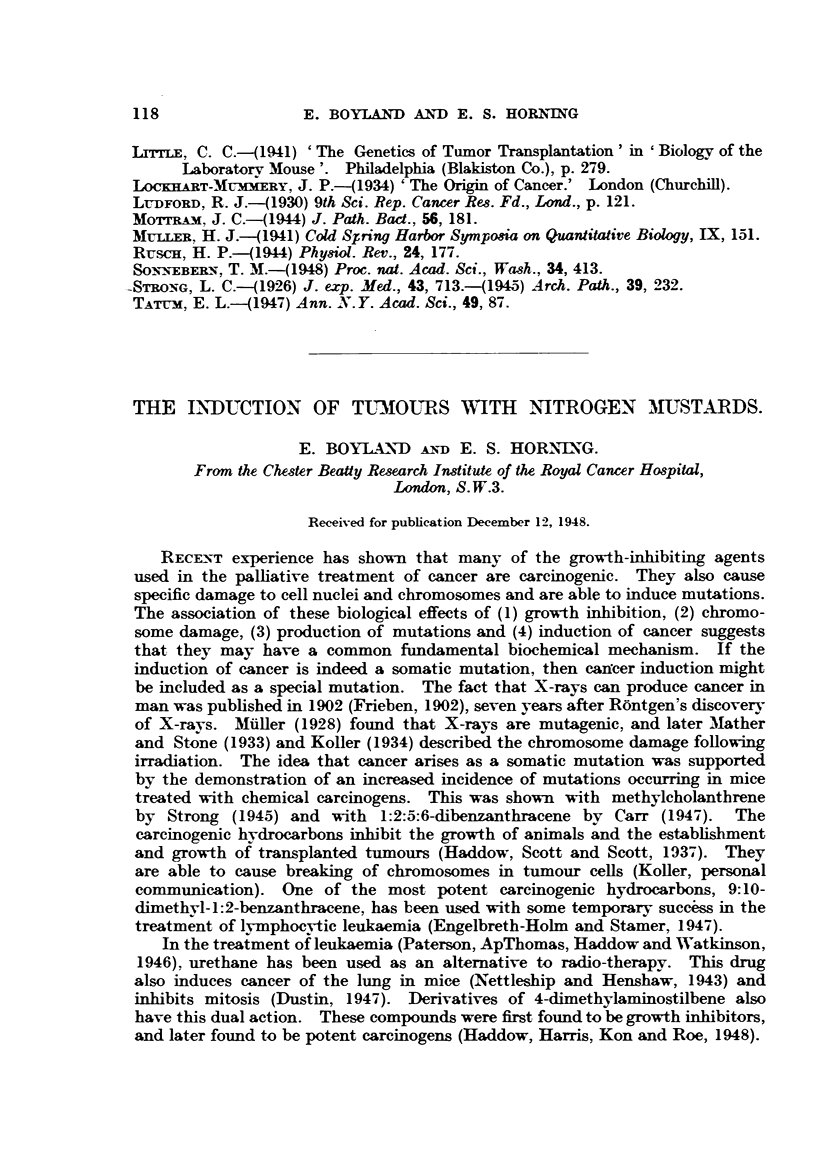# An Experimental Study of the Initiating Stage of Carcinogenesis, and a Re-examination of the Somatic Cell Mutation Theory of Cancer

**DOI:** 10.1038/bjc.1949.13

**Published:** 1949-03

**Authors:** I. Berenblum, P. Shubik


### AN EXPERIMENTAL STUDY OF THE INITIATING STAGE OF CARCINOGENESIS, AND A RE-EXAMINATION OF THE SOMATIC CELL MUTATION THEORY OF CANCER.

I. BERENBLUM AND P. SHUBIK.

*From the Oxford University Research Centre of the British Empire Cancer Campaign, Sir William Dunn School of Pathology, University of Oxford.*

Received for publication November 20, 1948.

THOUGH non-carcinogenic to normal skin, croton oil elicits tumours in skin previously treated with a carcinogen for an inadequate period (Berenblum, 1941). This procedure, with the modification of Mottram (1944), using only one single application of carcinogen prior to the croton oil treatment, served as the basis of a quantitative analysis of carcinogenic response (Berenblum and Shubik, 1947*b*). The results led to the establishment of the essential difference in mechanism between the preliminary " initiating process " and the subsequent " promoting process " of carcinogenesis. For reviews of the literature dealing with earlier work on the stages of carcinogenesis, and with the previous terminologies used, see Berenblum, 1944, 1947 ; Rusch, 1944 ; Berenblum and Shubik, 1947*a* and *b*.

From this analysis (Berenblum and Shubik, 1947*b*), it was concluded that the initiating process represents a sudden and irreversible change in a small minority of the cells of the treated area, giving rise to isolated " latent tumour cells," apparently indistinguishable morphologically from the surrounding non-neoplastic cells. The presence of these latent tumour cells is only demonstrable by subsequent promoting action, which converts them into morphological tumours. This promoting action is less specific than the initiating action, in that it can as readily be induced by croton oil as by continued applications of a true carcinogen. The irreversible nature of the initiating process was demonstrated by the fact that the number of tumours elicited by croton oil was as great after an interval of 20 weeks (between the carcinogen application and the commencement of croton oil treatment) as after an interval of only 3 days.

According to this new concept, the latent period of carcinogenesis is dependent on the efficacy of the promoting action, while the actual tumour yield is predetermined by the initiating action. This was borne out by comparative tests, using 1:2:5:6-dibenzanthracene, 3:4-benzpyrene, and 9:10-dimethyl-1:2-benzanthracene, as initiators, followed by croton oil treatment. It was found, as expected, that whereas the percentage of tumour-bearing animals differed in the three series, the latent periods were approximately the same.

Unfortunately, owing to considerations of solubilities, it was necessary, in these comparisons, to use a different concentration for each carcinogen. Consequently, more than one variable was involved in the experiment. While the results obtained provided an adequate basis for the general conclusions referred to above, it was felt, nevertheless, that unequivocal evidence could best be provided by studying the effects of a series of graded concentrations of one and the same carcinogen as initiator. For this investigation, described below, 9:10-dimethyl-1:2-benzanthracene was chosen as initiator.

An additional purpose of this investigation was that the quantitative data thus obtained could, at the same time, serve as indirect evidence for or against the possibility of a " mutation-like change " for the initiating stage of carcinogenesis. However, for a re-assessment of the validity of the " somatic cell mutation theory of cancer " in the light of the dual-stage mechanism of carcinogenesis, a more direct experimental approach was required. Such an approach seemed possible from the following considerations.

Mustard gas is known to possess mutagenic properties when tested on Drosophila (Auerbach and Robson, 1946, 1947), and on Neurospora and *E. coli* (Tatum, 1947), so that, according to the somatic cell mutation theory, this compound should act as a carcinogenic initiator. Admittedly, mustard gas is a potent anti-carcinogenic agent (Berenblum, 1929), but this effect was shown to be confined to the later stages of carcinogenesis (Berenblum, 1929, 1931), and could thus be attributed to an anti-promoting action; thus, mustard gas should, theoretically, still be capable of initiating action. For a histological study of the effects of low concentrations of mustard gas on the mouse's skin, and the resemblance of the changes produced to those of a single massive dose of X-rays, see Fell and Allsopp, 1948.

Mustard gas was, therefore, tested for initiating action, under the standard experimental conditions, using croton oil as the promoting agent.

### METHODS.

White female mice of the Swiss strain, originating from the Medical Research Council stock and since bred in this laboratory, were used for these experiments. The exclusive use of female mice eliminated the complication of skin irritation through fighting, the animals being kept in groups of 25 in large metal cages. The mice were maintained on an adequate mixed diet, with water *ad lib.* The experimental areas of skin, in the inter-scapular regions, were clipped periodically with fine scissors for removal of hair. All test solutions were applied with a glass rod, and colourless, non-fluorescent, liquid paraffin (" liquid Petrolatum ") was used as solvent throughout, for standardization of response (Berenblum and Schoental, 1947; Berenblum and Shubik, 1947*a*).

The main departure from the technique of the previous experiments was that instead of merely noting the numbers of tumour-bearing mice, the individual tumours were also recorded. The method adopted was to number each mouse by ear punching as soon as a tumour appeared, and to chart that tumour, and all subsequent ones, at fortnightly intervals throughout the experiment. Thus, the results are expressed in terms both of total numbers of tumours induced and of numbers of tumour-bearing animals, the former being also expressed in a corrected form, to exclude those " tumours " which regressed within 2 weeks of their appearance, whose neoplastic nature was obviously in doubt, and never confirmed.

The tumours produced were all papillomas in the first instance, judged by naked-eye evidence, and in many cases confirmed by histological examination after death. These papillomas appeared to behave very much like those induced by repeated painting with carcinogens, with a tendency to grow in size, and, in many cases, to become carcinomas, but also with a tendency for some of the papillomas to regress after a period of progressive growth. There was only one exception, in which the malignant tumour induced had the histological character of a sarcoma.

### EXPERIMENTAL.

*Comparison of graded concentrations of initiator.*

Four groups of 100 mice each, were used for this investigation. These received a single painting of 9:10-dimethyl-1:2-benzanthracene in liquid paraffin, to serve as initiator. Four weeks later, the same area of skin was submitted to twice-weekly applications of croton oil in liquid paraffin for 25 weeks, for promoting action.

The concentration of initiator solution varied in the 4 groups as follows :

Group AG, 0·06 per cent ; Group AA, 0·17 per cent ; Group AB, 0·5 per cent ; Group AC, 1·5 per cent.

The croton oil solution used was 5 per cent (except that a 10 per cent solution was used for 25 mice of each group for the first 7 weeks. Since the results obtained were, in each case, essentially the same as in the remaining 75 mice, the figures were combined). A control group of 25 mice which received a single application of 9:10-dimethyl-1:2-benzanthracene, without subsequent croton oil treatment, failed to yield any tumour.

TABLE I.—*The Potency of 9:10-Dimethyl-1:2-benzanthracene as Initiator, Tested in Different Concentrations, when Applied Once Only to the Mouse's Skin, Followed by Repeated Croton Oil Treatment.*

| Series. | Concentration of carcinogen. %. | Latent period (weeks). | Number of mice with tumours. | Total number of tumours. | Average number of tumours per tumour-bearing animal. |
|---|---|---|---|---|---|
| AG | 0·06 | 14·4 | 9 | 10 | 1·1 |
| AA | 0·17 | 12·1 | 25 | 61 | 2·4 |
| AB | 0·5 | 10·6 | 43 | 118 | 2·7 |
| AC | 1·5 | 11·8 | 63 | 248 | 3·9 |

Duration of experiment : 29 weeks (i.e. 25 weeks from commencement of croton oil treatment).
Number of animals : 100 per group.

The results obtained are summarized in Table I, showing : (1) the average latent period per group (estimated on the basis of the appearance of the first tumour per animal) ; (2) the numbers of tumour-bearing mice ; (3) the total numbers of individual tumours, and (4) the average number of tumours per tumour-bearing animal.

It will be noted that, with the ascending increase in the concentration of initiator, there was a progressive increase in the tumour yield, both as expressed in terms of tumour-bearing animals per group, and, more strikingly, in terms of individual tumours produced ; while the average number of tumours per tumour-bearing animal also increased (from 1·1, for the lowest concentration, to 3·9 for the highest). Nevertheless, the average latent periods showed no significant variation in the 4 groups. These results may be taken as a clear and striking confirmation of the concept (Berenblum and Shubik, 1947*b*) that the tumour incidence is directly dependent on the initiating action, while the latent period is a function of the promoting action.

TABLE II.—*A Comparison of Ratios of the Results Obtained* (*Table I*).

| Series. | Ratios of: Concentration of carcinogen. | Numbers of tumour-bearing mice. | Total numbers of tumours. | Total numbers of tumours, less early regressions. |
|---|---|---|---|---|
| AG | 1 | 1·0 | 1·0 | 1·0 |
| AA | 3 | 2·8 | 6·1 | 6·6 |
| AB | 9 | 4·8 | 11·8 | 12·4 |
| AC | 27 | 7·0 | 24·8 | 26·5 |

When the results are compared in terms of ratios (Table II), it becomes apparent that the actual tumour yield approximately bears a direct 1:1 relation to the concentration of the initiator (thus, with a 27-fold increase in concentration of initiator, there was a 26·5-fold increase in tumour yield). Its significance in relation to the somatic cell mutation theory of cancer will be discussed below.

*Tests of mustard gas for initiating action.*

Two groups of 25 mice were used for this investigation, one receiving a single, and the other 3 weekly, applications of a 0·1 per cent solution of mustard gas in liquid paraffin. Both groups were then left untreated for 3 weeks, and thereafter the experimental area of the skin was painted twice weekly for 25 weeks with a 5 per cent solution of croton oil in liquid paraffin.

Except for 3 small nodules, at first believed to be early papillomas, which regressed within 1–4 weeks, no tumours appeared in either group.

### DISCUSSION.

It was previously shown (Berenblum and Shubik, 1947*b*) that by varying the nature and concentration of carcinogen as initiator (applied once only), followed by repeated croton oil applications, different tumour yields were obtained, though the average latent periods for the different groups remained approximately the same. From these results, and from other evidence brought forward, it was concluded that the ultimate tumour yield in carcinogenesis is pre-determined by an irreversible initiating process, while the latent period of carcinogenesis, i.e. the speed with which the induced "latent tumour cells" are converted into morphological tumours, is a function of the subsequent promoting process.

The idea of the possible existence of "latent tumour cells" requiring additional stimulation, i.e. promoting action, for their conversion into morphological tumours, arose from the work of Friedewald and Rous (1944) on the disappearance and reappearance of carcinogenically-induced papillomas in rabbits. The experiments on mice with croton oil (Berenblum and Shubik, 1947*b*) provided the quantitative data for the establishment of the two-stage mechanism of carcinogenesis, with "latent tumour cells" as the product of the first, or initiating, stage. A similar belief in the existence of "latent tumour cells" as the basis of tumour production, has also been expressed by Fischer (1937), as a consequence of his studies on tumour development following the repeated transplantation of normal mammary tissue in series, in mice.

An interesting confirmation of the two-stage mechanism of carcinogenesis has recently become available (Hall, 1948), from a follow-up of the work of Bielschowsky (1945, 1947) on tumour production in the thyroid gland by the combined action of 2-acetylaminofluorene and goitrogenic agents. Hall studied the effect of the goitrogenic agents before and after a period of treatment with acetylaminofluorene, and found that augmentation of tumour induction only occurred when these were administered after the acetylaminofluorene. The effect was still observed when there was a long interval between the two treatments. The results suggest that the acetylaminofluorene acted as initiator, and the goitrogenic agent as promoter, in an analogous fashion to the carcinogen and the croton oil, respectively, in the case of skin experiments.

In the present investigation, one and the same carcinogen was applied to the mouse's skin, once only in different (graded) concentrations, to serve as initiator, and then followed by repeated applications of croton oil. Expressed in ratios, the concentrations of initiator (9:10-dimethyl-1:2-benzanthracene in liquid paraffin) in the four experimental groups were 1:3:9:27, and the total yields of tumours for the corresponding groups were in the ratio of 1:6·6:12·4:26·5 (Table II). Yet the average latent periods for these 4 groups were closely similar, namely, 14·4, 12·1, 10·6, and 11·8 weeks, respectively. Not only was there a progressive increase in the total number of tumours, with increase in concentration of initiator, and also in the number of tumour-bearing animals, but the actual number of tumours per tumour-bearing animal rose progressively, from an average of 1·1, in the lowest concentration, to 3·9 in the highest.

These results provide a striking confirmation of the concept outlined above concerning the two-stage mechanism of carcinogenesis.

The evidence that the initiating process of carcinogenesis is sudden and irreversible would seem to suggest a "mutation-like" change, and the indication that the change affects very few of the cells in the treated area (possibly even a single cell per locus) also supports this supposition. It became necessary, therefore, to re-examine the somatic cell mutation theory of cancer in the light of this new concept.

This theory, first dealt with at length by Bauer (1928), and reviewed by Ludford (1930), Lockhart-Mummery (1934), and many others, considers neoplasia to be the consequence of a changed gene. It can, by its very nature, only be submitted to verification of an indirect kind (Haldane, 1934), since the only proof of a mutation is that achieved by crossing the mutated cell with a normal cell. In the case of a somatic cell, which cannot conjugate, this is manifestly impossible. Many different experimental approaches have, however, been devised in recent years to obtain, at least, indirect evidence in support of the theory, and these may conveniently be considered under the following headings :

1. *Induction, by known carcinogenic agents, of mutations in lower organisms.*

The early studies in this field were concerned with X-rays and ultraviolet radiation, the literature of which is well reviewed by Muller (1941). Although most of this work was not directly concerned with correlating carcinogenicity and mutagenic potency, the fact that an apparent association, in the case of physical agencies, did exist, was widely accepted as strong evidence in support of the somatic cell mutation theory of cancer.

The discovery of chemical mutagens (e.g. mustard gas) by Auerbach and Robson (1946, 1947), helped to extend the field of enquiry, and made possible detailed comparisons between carcinogenic and mutagenic activity of various compounds, especially among the polycyclic hydrocarbons.

Tatum (1947), using neurospora as indicator, studied various types of mutations induced with 20-methylcholanthrene, but, while reviewing the somatic cell mutation theory, he did not make quantitative correlations between the two properties possessed by this compound. Using various carcinogenic and non-carcinogenic hydrocarbons in the form of aerosols, tested on drosophila, Demerec (1948) was able to find some correlation between carcinogenicity and the power to produce mutation, though the correlation was not absolute.

Demerec and Latarjet (1946) had previously tested X-rays and ultraviolet radiations on bacteria, giving precise data in relation to mutagenic and lethal power. More recently, using a wide range of both water-insoluble (colloidal dispersions) and water-soluble carcinogenic and non-carcinogenic compounds, tested under similar conditions to those of Demerec (1948) and studied on a quantitative basis, Latarjet (1948) failed to find any correlation between mutagenic and carcinogenic potencies.

Briefly, therefore, the apparent correlation between mutagenic and carcinogenic activities, as noted in the case of physical agencies, has not been confirmed when extended to chemical agents, and can no longer be used as strong support in favour of the somatic cell mutation theory of cancer.

2. *Genetic analysis of tumour transplantation phenomena in pure strain mice.*

The genetic control of tumour transplantation in pure strain mice has been investigated in some detail, and the earlier work in this field is well summarized by Little (1941). It has been shown that a number of genes control the behaviour of certain tumours, and this approach has been extended further by Gorer (1937, 1947), showing that these genes probably determine their antigenic properties. Also, according to Strong (1926), Bittner (1931) and others, the observed sudden change in the transplantation properties of tumours could be explained on the basis of the occurrence of mutations. The comparison between transplantation properties of normal and tumour tissues, originally suggested by Little (1941), was undertaken by Furth, Boon and Kaliss (1944), who made such comparisons between certain transplantable tumours and leukaemias against normal spleen, and found the transplantation behaviour to be different in the two classes of tissues, thus pointing to a difference in their genetic make-up. From this they concluded that a somatic mutation would be a more likely explanation of the changeover from the normal to the neoplastic state than any type of " differentiation."

It must be remembered, however, that differences in transplantation behaviour involve many so-far unexplained anomalies, some of which have been forcefully brought out by the work of Greene (1944), and that analogies between the genetics of transplantation (with all the complications of adaptability in another host) and that of tumour induction (where the autologous nature of the tumour cells is self-evident) may be more complex than is generally supposed. This type of evidence cannot, therefore, be considered as more than suggestive in support of the somatic cell mutation theory.

3. *Induction of germinal mutations in mice by means of carcinogens.*

The work of Strong (1945) on the development of germinal mutations in mice injected with methylcholanthrene, led him to suggest that somatic mutations might well be engendered by the same agency. Carr (1947) has also claimed to have produced such germinal mutations with 1:2:5:6-dibenzanthracene. The validity of the results have been questioned (Heston, 1948).

So far, the number of germinal mutations attributed to carcinogenic hydrocarbons has been very small, and further work along these lines is clearly needed, in order to determine whether carcinogenic hydrocarbons are, indeed, more effective than other compounds in inducing germinal mutations. Until this is known, it is difficult to decide how far these results are significant as evidence in favour of the somatic cell mutation theory.

4. *Quantitative interpretation of carcinogenic experiments.*

Several attempts have been made to evaluate quantitatively some of the more reliable carcinogenic data, to determine whether or not such data would be in keeping with a sudden irreversible change of a mutation-like nature.

In the investigation of Dunning, Curtis and Wood (1940), the production of sarcomas in rats, by subcutaneous injection of 3:4-benzpyrene, was submitted to quantitative analysis. The concentration of carcinogen and the volume of fluid injected, both of which were variables, were correlated with the number of tumours induced and with the latent period of tumour induction. It was found that increasing the number of foci injected did increase the number of tumours induced, whereas increasing the volume of fluid injected in one locus did not. In view of the variables involved, and for other reasons to be discussed later, these results do not lend themselves to an exact interpretation of a single response to a single stimulus.

Charles and Luce-Clausen (1942) analysed data of a mouse skin carcinogenesis experiment, using continued painting with 3:4-benzpyrene, to determine whether or not this was in keeping with the concept of the somatic cell mutation theory. In their introduction, they make the primary assumption that if a mutation were the basis of carcinogenesis, it must, of necessity, be of a recessive nature, thus postulating two successive mutations to bring to light an actual tumour. There is, however, no valid reason for this assumption, which is, in fact, at variance with the theory of dominancy, as proposed by Fisher (1930).

In all these previous attempts at analysing carcinogenic experiments, in which the carcinogen is allowed to act continuously, there is the inevitable complication arising from the fact that, of the two stages of carcinogenesis, only one—the initiating stage—could possibly be ascribed to a mutation, since this is the stage which involves a sudden and irreversible change, possibly affecting one cell only. A true quantitative analysis, bearing on the mutation theory, can only be acquired by examining the carcinogen in an uncomplicated light, i.e. as initiator only, and, if possible, on one occasion only in this role.

In the present investigation, the correlation of concentration of carcinogen, as initiator, with the total number of tumours induced, has revealed approximately a direct 1:1 ratio. This could be taken as being in keeping with the somatic cell mutation theory.

However, the other investigation, described above, does not lend support to this theory. As already mentioned, mustard gas is a potent mutagenic agent on drosophila (Auerbach and Robson, 1946, 1947). Since its anti-carcinogenic properties (Berenblum, 1929, 1931) can undoubtedly be attributed to anti-promoting action, it was to be expected, according to the somatic cell mutation theory, that when allowed to act once only on mouse's skin, followed by repeated croton oil treatment, tumours should have arisen, the mustard gas acting as an initiator by virtue of its mutagenic properties. When tested, this was found not to be the case.

In view of this negative finding, and in the light of the highly conflicting evidence of recent experiment in an attempt to adduce more indirect support of the theory, it becomes necessary to re-assess the validity of the theory.

Basically, neither proof nor disproof of the somatic cell mutation theory of cancer is possible (Haldane, 1934), as referred to above. The accumulating indirect evidence would seem to run counter to the theory instead of supporting it. Of the evidence presented in the present communication, the first experiment is consistent with the theory, while the second is strongly against it.

The force of past reasoning in support of the theory has rested largely on the assumption that, given an irreversible change as the basis of carcinogenesis, the only known biological phenomenon to explain this would be a mutation. However, a closer examination of other common biological phenomena instantly reveals that this is not so. For example, in the course of the development of the embryo, the divergent differentiation that ultimately results in the irreversible cell types, e.g. into epithelial, nerve, and muscle cells, etc., represents just such a non-mutational irreversible process. Moreover, from the early work of Cohnheim, it is possible that aberrant embryonic differentiation may occur, though this probably provides no more than a partial explanation of certain rare and very specialized groups of tumours.

The substitution of a concept of non-mutational "aberrant differentiation," divorced from Cohnheim's all-embracing embryological implications, in the place of a somatic mutation mechanism, to account for the initiating process of carcinogenesis, clearly deserves further consideration. It is tempting to draw a parallel, from the knowledge of embryological development, between the "organizer" and the "latent tumour cell," on the one hand, and between the "evocator" and the "carcinogenic promoting agent" on the other. In our present state of knowledge, such a hypothesis would, admittedly, be a dangerous over-simplification; nevertheless, as a guide for further investigation, the analogy may possibly serve a useful purpose. On the question of the mechanism of non-mutational, hereditable transmission of new characters, the action of viruses, particularly the example of the milk-factor in the development of mammary carcinoma in mice, Bittner, 1936, 1939, and the recent work of Sonneborn (1948), on the transmission of antigenic and other differences in genetically identical paramecium cells, should also be taken into account.

In conclusion, whatever interpretation is adopted as a base-line for research, the recognition that carcinogenesis is at least a two-stage process, should invariably be borne in mind. Any basic theory that overlooks this must, of necessity, be incomplete.

SUMMARY.

1. With the standard experimental technique, using a single application of a carcinogen followed by repeated paintings of the same area of skin with croton oil, a quantitative correlation has been made between the concentration of the carcinogen (initiating agent) and the total number of tumours induced.

2. It was found that there exists approximately a direct 1:1 ratio between the two.

3. The known mutagenic agent—mustard gas—was investigated under the conditions of the standard experiment, as initiating agent, and found to be negative.

4. The results are discussed in relation to the somatic cell mutation theory of cancer.

We wish to thank Mr. H. W. Wheal for technical assistance and the care of the animals.

REFERENCES.

AUERBACH, C., AND ROBSON, J. M.—(1946) *Nature*, **157**, 302.—(1947) *Proc. Roy. Soc. Edinb.*, **62**, 271.

BAUER, K. H.—(1928) 'Mutationstheorie der Geschwulst-Enstehung.' Berlin (Julius Springer).

BERENBLUM, I.—(1929) *J. Path. Bact.*, **32**, 425.—(1931) *Ibid.*, **34**, 731.—(1941) *Cancer Res.*, **1**, 807.—(1944) *Arch. Path.*, **38**, 233.—(1947) *Brit. med. Bull.*, **4**, 343.

*Idem* AND SCHOENTAL, R.—(1947) *Cancer Res.*, **7**, 390.

*Idem* AND SHUBIK, P.—(1947*a*) *Brit. J. Cancer*, **1**, 379.—(1947*b*) *Ibid.*, **1**, 383.

BIELSCHOWSKY, F.—(1945) *Brit. J. exp. Path.*, **26**, 270.—(1947) *Brit. med. Bull.*, **4**, 382.

BITTNER, J. J.—(1931) *Amer. J. Cancer*, **15**, 2202.—(1936) *Science*, **84**, 162.—(1939) *Amer. J. Cancer*, **35**, 90.

CARR, J. G.—(1947) *Brit. J. Cancer*, **1**, 152.

CHARLES, D. R., AND LUCE-CLAUSEN, E. M.—(1942) *Cancer Res.*, **2**, 261.

COHNHEIM, J.—(1889) 'Lectures on General Pathology.' London (The New Sydenham Society).

DEMEREC, M.—(1948) *Brit. J. Cancer*, **2**, 114.

*Idem* AND LATARJET, R.—(1946) *Cold Spring Harbor Symposia on Quantitative Biology* XI, 38.

DUNNING, W. F., CURTIS, M. C., AND WOOD, F. C.—(1940) *Amer. J. Cancer*, **39**, 70.

FELL, H. B., AND ALLSOPP, C. B.—(1948) *Cancer Res.*, **8**, 177.

FISCHER, A.—(1937) *Amer. J. Cancer*, **31**, 1.

FISHER, R. A.—(1930) 'The Genetical Theory of Natural Selection.' London (Oxford University Press).

FRIEDEWALD, W. F., AND ROUS, P.—(1944) *J. exp. Med.*, **80**, 101.

FURTH, J., BOON, M. C., AND KALISS, N.—(1944) *Cancer Res.*, **4**, 1.

GORER, P. A.—(1937) *J. Path. Bact.*, **44**, 691.—(1947) *Cancer Res.*, **7**, 634.

GREENE, H. N. S.—(1944) *Amer. Ass. Adv. Sci. Res. Conf. Cancer*, 154.

HALDANE, J. B. S.—(1934) *J. Path. Bact.*, **38**, 507.

HALL, W. H.—(1948) *Brit. J. Cancer*, **2**, 273.

HESTON, W. E.—(1948) *Advances of Genetics*, **2**, 120.

LATARJET, R.—(1948) *C. R. Soc. Biol., Paris*, **142**, 453.

LITTLE, C. C.—(1941) 'The Genetics of Tumor Transplantation' in 'Biology of the Laboratory Mouse'. Philadelphia (Blakiston Co.), p. 279.
LOCKHART-MUMMERY, J. P.—(1934) 'The Origin of Cancer.' London (Churchill).
LUDFORD, R. J.—(1930) *9th Sci. Rep. Cancer Res. Fd., Lond.*, p. 121.
MOTTRAM, J. C.—(1944) *J. Path. Bact.*, **56**, 181.
MULLER, H. J.—(1941) *Cold Spring Harbor Symposia on Quantitative Biology*, IX, 151.
RUSCH, H. P.—(1944) *Physiol. Rev.*, **24**, 177.
SONNEBERN, T. M.—(1948) *Proc. nat. Acad. Sci., Wash.*, **34**, 413.
STRONG, L. C.—(1926) *J. exp. Med.*, **43**, 713.—(1945) *Arch. Path.*, **39**, 232.
TATUM, E. L.—(1947) *Ann. N.Y. Acad. Sci.*, **49**, 87.

---

### THE INDUCTION OF TUMOURS WITH NITROGEN MUSTARDS.

E. BOYLAND AND E. S. HORNING.